# Controlled clinical trial addressing teeth whitening with hydrogen peroxide in adolescents: a 12-month follow-up

**DOI:** 10.6061/clinics/2017(03)06

**Published:** 2017-03

**Authors:** Marcelo Mendes Pinto, Marcela Leticia Leal Gonçalves, Ana Carolina Costa da Mota, Alessandro Melo Deana, Silvia Regina Olivan, Carolina Bortoletto, Camila Haddad Leal de Godoy, Katia Lumi Tanikawa Vergilio, Olga Maria Altavista, Lara J Motta, Sandra Kalil Bussadori

**Affiliations:** Universidade Nove de Julho, Programa de Pós-Graduação, Departamento de Biofotônica Aplicada às Ciências da Saúde, São Paulo/SP, Brazil

**Keywords:** Tooth Bleaching, Adolescent, Tooth Bleaching Agents

## Abstract

**OBJECTIVES:**

To evaluate the colorimetric change in incisors and canines of adolescents aged 12 to 20 years submitted to at-home whitening and to evaluate satisfaction, sensitivity and discomfort during the procedures through a questionnaire.

**METHOD:**

Thirty adolescents were randomly assigned to the following groups: 1) 6.0% hydrogen peroxide (White Class with calcium – FGM); 2) 7.5% hydrogen peroxide (White Class with calcium - FGM); 3) 10% hydrogen peroxide (Oral B 3D White – Oral-B); 4) Control group – placebo. Assessments were performed prior to treatment as well as at 7, 30, 180 and 360 days after treatment. Friedman’s ANOVA was used to analyze color. The Kruskal-Wallis test followed by Dunn’s post hoc test was used to compare the groups at the different evaluation times. Answers on the questionnaires were ranked, and non-parametrical tests were employed. The groups were compared in each period using the Kruskal-Wallis test followed by the Student-Newman-Keuls test. Categorical data were analyzed using Fisher's exact test, and the Wilcoxon test was used for the analysis of different periods. P-values were corrected using the Hyan-Holm step-down Bonferroni procedure. Clinicaltrials.gov: NCT01998386.

**RESULTS:**

Similar results were obtained one month after treatment with both tooth whitening gels and whitening strips. Patients were partially satisfied with the treatment after the first and second weeks and would recommend it. All products demonstrated color stability after 12 months of follow-up.

**CONCLUSIONS:**

The bleaching procedure was efficient, and the patients could perceive its result. Further investigations are needed to determine the effects of bleaching on young teeth.

## INTRODUCTION

Adolescents and young adults are increasingly concerned about dental esthetics, which may be one of the factors that has led to the demand for products to improve tooth color 1. The American Academy of Pediatric Dentistry has recognized the increased interest of young people in whitening their teeth, which is likely due to the increase in the variety, availability and popularity of whitening products 1. Tooth whitening is considered the least invasive esthetic procedure for yellowed teeth and the most sought-after type of treatment among patients 2.

Over-the-counter tooth whitening products emerged in the USA at the beginning of the century as a treatment option for tooth stains at a lower cost than traditional treatment performed by a dentist 3. In the last ten years, whitening agents have been added to over-the-counter products, such as toothpastes, mouthwashes and chewing gum 4. While the quantity of such products has increased considerably since their appearance on the market, the concentrations of peroxide are so low that one may question their actual whitening potential 5,6. Moreover, it should be stressed that such materials are generally employed with no follow-up or technical supervision, raising concerns in the scientific community. This is especially true when considering the abrasiveness of these products and their possible morphological changes and negative effects, such as increased toothsensitivity and compromised enamel structure 4,7-10. Furthermore, patients with severe discoloration, such as tetracycline staining and fluorosis, may believe that the products will solve their problem instead of seeking professional help.

At-home tooth whitening with a gel and plastic mold supervised by a dentist is the most widely employed method 11. For this treatment, 10% carbamide peroxide is delivered in a custom-fitting mouth tray. The method was introduced by Klusmier in the late 1960s 12, published by Haywood and Heymann in 1989 and has become the gold-standard treatment in tooth whitening 3.

As teenagers are very concerned with the social aspect of their appearance, it is important to evaluate the efficiency of the bleaching procedure in this population and patient satisfaction after treatment. Moreover, body dysmorphic disorder (BDD) (a condition in which people are obsessed with their appearance) is highly prevalent in adolescents and cosmetic dentistry settings 12. Sensitivity is also an important matter since it affects 8 to 66% of patients and usually occurs in the early stages of treatment 14-16. It is also necessary to assess the discomfort and difficulty of a technique to determine whether it is appropriate for a particular patient. Previous studies have employed questionnaires as a good way to evaluate the level of satisfaction, sensitivity and discomfort during and after procedures 16.

Long-term controlled clinical trials are needed to evaluate the use of over-the-counter products, such as whitening strips, in comparison to other self-administered methods, especially among adolescents. Thus, the aim of the present randomized, controlled, clinical trial was to evaluate the colorimetric change on the incisors and canines of adolescents aged 12 to 20 years submitted to tooth whitening with 6 and 7.5% hydrogen peroxide using at-home methods (gel + mouth tray and whitening strips) as well as to evaluate the satisfaction, sensitivity and discomfort during and after the procedures through the use of a questionnaire.

## METHODS

This study received approval from the Human Research Ethics Committee of the University Nove de Julho (São Paulo, Brazil) under process number 410.582 and is registered with Clinical Trials under the number NCT01998386 (clinicaltrials.gov). Informed consent was obtained from all individual participants included in the study or from their guardians.

Thirty adolescents aged 12 to 20 years were selected and randomly assigned to three groups submitted to different commercially available tooth whitening products: 1) 6.0% hydrogen peroxide (White Class with calcium – FGM); 2) 7.5% hydrogen peroxide (White Class with calcium - FGM); and 3) 10% hydrogen peroxide (Oral B 3D White – Oral-B) (Chart 1). Ten adolescents (Group 4) were allocated to a control group to receive a placebo gel before being placed randomly into one of the other groups. Patients with their complete permanent dentition were included in the study, while patients with periodontal diseases, cavities or severe discolorations or those undergoing orthodontic treatment were excluded.

**Figure 1:**
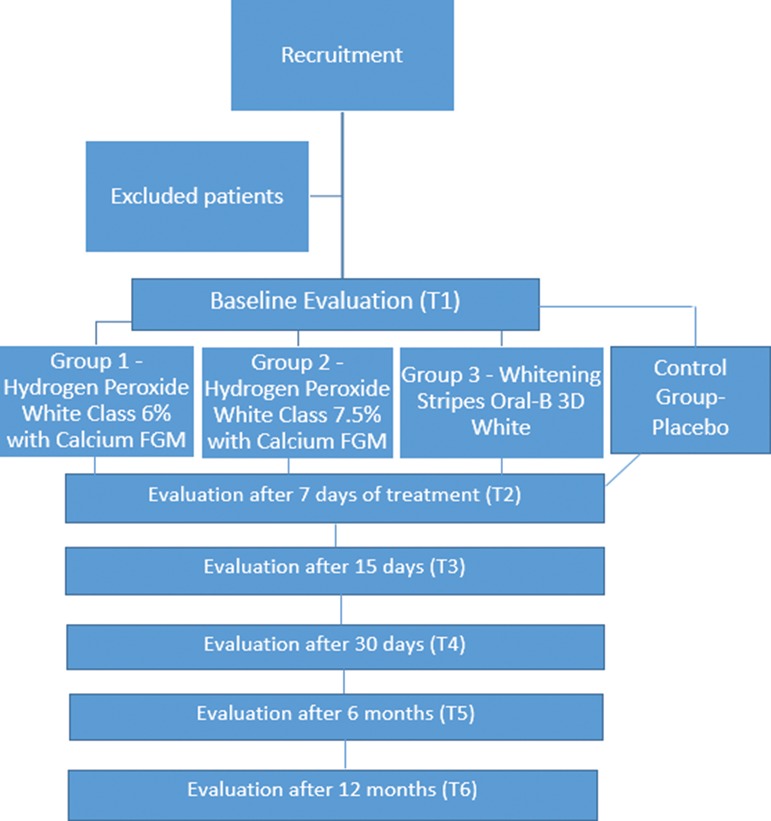
Flowchart of the study based on CONSORT 17 recommendations.

The whitening products were used for seven days. During the treatment, patients were instructed not to drink or eat anything too pigmented. Assessments were performed prior to treatment as well as 7, 30, 180 and 360 days after treatment (Figure 1). The initial and follow-up color values on the incisors and canines were determined using the Vita Toothguide 3D-Master color scale (VITA). After treatment, each subject was administered a questionnaire that asked a set of questions addressing the difficulty, discomfort and satisfaction with the procedure.

### Statistical analysis

#### Color assessment

Friedman’s ANOVA was used to analyze the color of each sample in each group at 7, 15, 30, 180 and 360 days. The Kruskal-Wallis test followed by Dunn’s post hoc test was used to compare the different groups at each evaluation time.

#### Analysis of questionnaires

The answers from the questionnaires were ranked for inferential analysis. Questions 2 and 8, 3 and 9, 4 and 10 and 7 and 11 were combined and analyzed together. The different groups were compared in each period using the Kruskal-Wallis test followed by the Student-Newman-Keuls test. Categorical data were analyzed using the Fisher's exact test, and the analysis between different periods (7 and 15 days) was carried out using the Wilcoxon test. For multiple comparisons, the p-value was corrected using the Hyan-Holm step-down Bonferroni procedure.

## RESULTS

Table 1 shows the demographic data of all groups.

### Color assessment

Each sample was visually compared to the Vita 3D-Master color scale. Each value on the scale was then ranked, with lower ranks indicative of whiter samples and higher ranks indicative of darker samples (Table 2).

**Figure 2:**
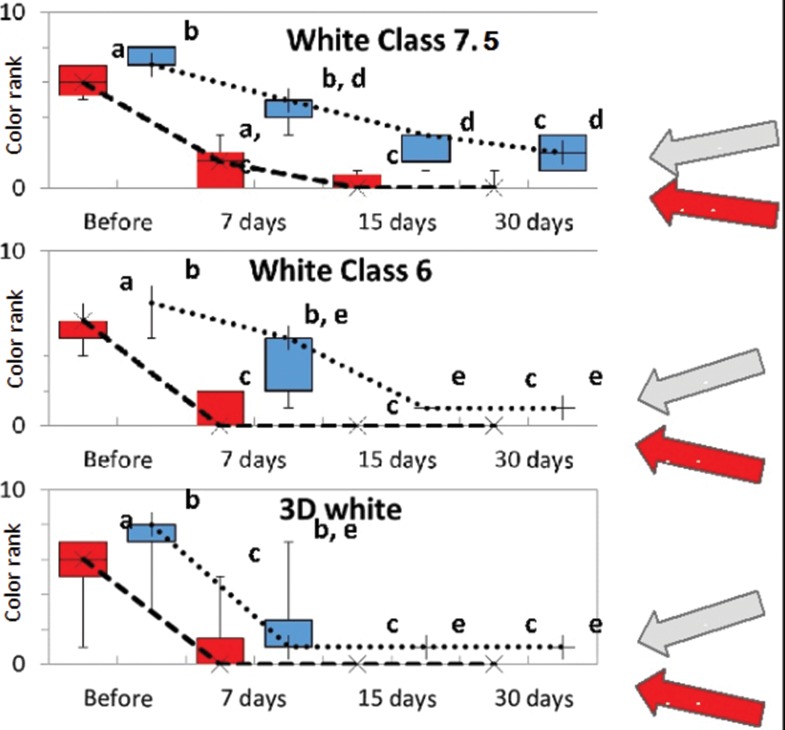
(color online) Boxplot of all groups: White class 7.5 (top), White class 6 (middle) and 3D White (bottom). Red boxes with ---X--- represent incisors, and blue boxes with ·····+····· represent canines. The same letters denote statistically equal color ranks.

Figure 2 shows that all groups had similar color ranks for the incisors and canines at the onset of the experiment (*p*=0.64 and *p*=0.34, respectively). After seven days of treatment, White Class 7.5 was not capable of significantly decreasing the median color rank (*p*=0.05 and *p*=0.15 for incisors and canines, respectively), whereas White Class 6 led to a significant reduction in median color rank for the incisors (*p*=0.03), but not the canines (*p*=0.09). 3D White also led to a significant reduction in color rank for the incisors (*p*=0.02), but not the canines (*p*=0.05). White Class 7.5 decreased the median color rank of the incisors to 1.5, and White Class 6 and 3D White decreased the median color rank to 0 after only seven days of treatment, with no significant difference among groups (*p*=0.63). One hundred percent of the individuals who used White Class 6 experienced a decrease in color rank to less than 2, and 75% of those who used 3D White experienced a decrease in color rank to less than 1, but this difference was not statistically significant after seven days of treatment. While White Class 7.5 and 6 also led to a decrease in the median rank color of the canines to 5 (2M3), 3D White led to a decrease to 1 within seven days of usage, but these differences were not statistically significant (*p*=0.09).

Fifteen days after treatment, White Class 7.5 led to a decrease in the median color rank of the incisors, ranging from 0 (1M1) to 1 (1M2) for the incisors and 0 to 3 (2M2) for the canines, which differed significantly from the values prior to treatment (*p*=0.005 and 0.002 for incisors and canines, respectively), but these results did not differ from those found at the Day 7 assessment (*p*=0.43 and 0.17 for incisors and canines, respectively). White Class 6 and 3D White also achieved significant differences in comparison to the pretreatment values for incisors and canines (White Class 6: *p*=0.004 and *p*=0.002, respectively; 3D White: *p*=0.005 and *p*=0.008, respectively). Although the median color rank for these whiteners was statistically unaltered in comparison to the Day 7 assessment of the incisors and canines (White Class 6: *p*=0.43 and *p*=0.17, respectively; 3D White: *p*=0.56 and *p*=0.47, respectively), both products led to a decrease in color rank among all subjects to 0 and 1 for incisors and canines, respectively, regardless of the initial conditions. The median color rank of the incisors did not differ statistically at 15 days (*p*=0.41); however, *p*=0.007 was found when comparing White Class 7.5 to White Class 6, and *p*=0.007 was found when comparing White Class 7.5 to 3D White for the canines.

White Class 7.5 required 30 days of treatment to stabilize the color rank of the teeth. The median color rank for the canines remained at 2 (2M1), with subjects ranging from 1 to 3 (1M2 - 2M2), while the median rank color for the incisors was 0 (1M1), with subjects varying up to 1 (1M2). Although, a small decrease was found in the median color rank 30 days after treatment, the difference did not achieve statistical significance in comparison to the Day 15 assessment (*p*=0.79 for both incisors and canines).

At the six-month and one-year assessments, the median color remained similar to that found at the Day 30 assessment in the White Class 7.5 group and the Day 15 assessment in the White Class 6 and 3D White groups. Thus, none of the groups darkened over time.

### Questionnaire

- Evaluation of the technique

Questions 2 and 8 aimed to assess the difficulty in placing the gel into the mold or the perception of difficulty in placing the strips on the teeth. All the subjects who used the techniques with molds (White Class 7.5, White Class 6 and control groups) found the application of the gel into the mold to be very easy to slightly difficult, but 30% of the strip group (3D White) found it difficult to correctly place the strip on the teeth. However, these differences were not statistically significant (*p*=0.16).

Regarding the overall difficulty of the entire technique (questions 3 and 9), on a scale of 0 (very easy) to 10 (very hard), all subjects in the mold groups (White Class 7.5, White Class 6 and control) rated it as 4 or lower, and 90% of the subjects in the White Class groups rated it as 3 or lower. These differences were not statistically significant (*p*=0.15). One subject in the 3D White group rated the overall technique as 5 and attributed his difficulty (question 10) to not knowing whether the strip was supposed to be placed on top or below, not knowing the position of the strip on the teeth as well as the lack of bonding of the strip to the gums and teeth.

With respect to the mold groups, 25 to 30% of all subjects needed to remove excess gel every time they performed the procedure, whereas 25 to 50% never needed to do so. These differences were not statistically significant (*p*=0.99). The majority of all groups chose rinsing as the preferable method to remove excess gel after the procedure, but some subjects chose to not remove the remaining gel regardless of the technique (*p*=0.359).

- Self-perceptions

The results concerning self-perceptions are shown in Table 3.

At the end of the first week, the overall complaints did not differ statistically between the White Class groups (*p*=0.12 and *p*=0.32 at 7 and 15 days, respectively), but complaints were significantly higher at the end of the first week in the 3D White group (*p*=0.003 and *p*<0.0001 in comparison to the White Class 7.5 and White Class 6 groups, respectively). At the end of the first week, the White Class 7.5 and 3D White groups also differed from the control group (*p*=0.02 and *p*<0.0001, respectively), but the White Class 6 group had statistically similar results (*p*=0.13). All groups were similar to the controls at the end of the second week.

**Figure 3:**
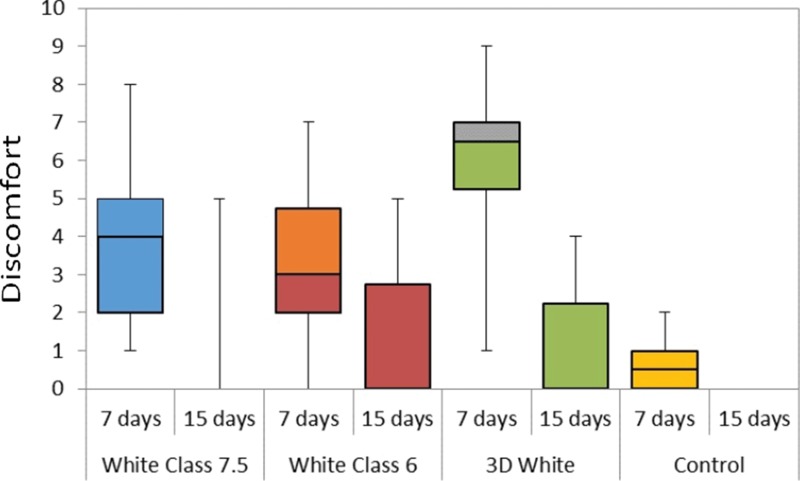
Discomfort grade of each group range: 0 (no discomfort) to 10 (extreme discomfort). The same letters indicate statistically equal groups.

Figure 3 shows the overall perception of discomfort by the subjects graded from 0 (no discomfort) to 10 (extreme discomfort).

At the end of the first week, discomfort in the White Class 6 was similar to that of the control group (*p*=0.06), but the White Class 7.5 and the 3D White groups exhibited significantly more discomfort than the control group (*p*=0.008 and *p*=0.0002, respectively). Discomfort was similar in all groups after 15 days (*p*=0.4492). Discomfort tended to decrease from the first to the second week. This decrease was significant in the White Class 7.5 (*p*=0.03) and 3D White (*p*=0.005) groups. To decrease discomfort, most subjects brushed more often, a few chose to rinse and none of the subjects stopped the procedure. In all groups, discomfort started a little earlier in the second week of treatment, but this was not statistically significant.

After the first week of the treatment, all groups had individuals who reported tooth sensitivity. In the second week, complaints decreased significantly only in the 3D White group (*p*=0.02; McNemar test). No significant differences in sensitivity were found among the groups submitted to whitening techniques in the analysis periods (*p*>0.05), but reports of sensitivity were greater in all groups when compared to the control group in the first week (*p*=0.009, *p*=0.0002 and *p*=0.0002 for White Class 7.5, White Class 6 and 3D White, respectively; Fisher’s exact test), as shown in Table 4.

Sensitivity usually began within two days of the onset of treatment, with the exception of the White Class 7.5 group, in which the only report of sensitivity in the second week began five days after the onset of the procedure. Differences among the groups submitted to whitening did not differ significantly (*p*>0.05; Kruskal-Wallis test). Among all subjects who reported sensitivity, only two complained in the first and second week, both of whom were from the White Class 7.5 group.

Regarding self-rated pain on a scale of 0 (no pain) to 10 (severe pain), 3D White was the only group that experienced a significant reduction in pain from the first to second week (*p*=0.02; Wilcoxon test). In the first week, median pain was similar among all treated groups, but it was significantly different in comparison to the control group (*p*=0.01, *p*=0.0006 and *p*=0.001 for the White Class 7.5, White Class 6 and 3D White groups, respectively; Student-Newman-Keuls test). In the second week, median pain was similar in all groups (*p*=0.60; Kruskal-Wallis).

**Figure 4:**
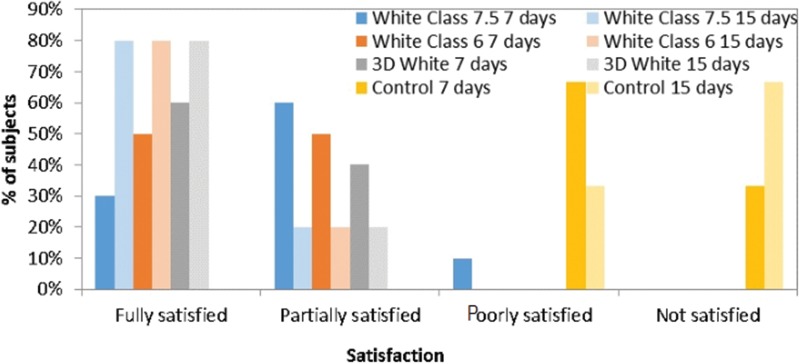
Subjects’ overall perception of satisfaction.

Most subjects who used White Class 6 and 3D White were fully satisfied with the overall results after only one week of whitening, with statistically significant differences in comparison to the control group (*p*=0.0001 and *p*<0.0001, respectively; Student-Newman-Keuls test). Similar results were found at the end of the second week, as all whitening groups differed statistically from the control (*p*=0.0033, *p*<0.0001 and *p*<0.0001 for White Class 7.5, White Class 6 and 3D White, respectively; Student-Newman-Keuls test), as shown in Figure 4. Pairwise comparisons demonstrated a lack of statistical significance for all groups when comparing results between the first and second weeks.

With regard to the self-rated overall satisfaction on a scale of 0 (not satisfied) to 10 (fully satisfied), the White Class 7.5 and 3D White groups experienced a significant improvement from the first week to the second week (*p*=0.005 and *p*=0.005, respectively; Wilcoxon test). In the first week, the median overall satisfaction was similar among all treated groups and differed significantly in comparison to the control group (*p*=0.0003, *p*<0.0001 and *p*<0.0001 for White Class 7.5, White Class 6 and 3D White, respectively; Student-Newman-Keuls test). In the second week, similar results were found (*p*=0.0002, *p*<0.0001 and *p*<0.0001 for the White Class 7.5, White Class 6 and 3D White groups, respectively; Student-Newman-Keuls test).

Most subjects who used whiteners found color differences after only one week of treatment, with statistically significant differences in comparison to the control group (*p*=0.0002, *p*=0.0002 and *p*<0.0001 for White Class 7.5, White Class 6 and 3D White, respectively; Student-Newman-Keuls test). Similar results were found in the second week, in which all whitening groups differed significantly from the control (*p*=0.0002, *p*=0.0002 and *p*<0.0001 for White Class 7.5, White Class 6 and 3D White, respectively; Student-Newman-Keuls test). No statistically significant differences were found between the first and second week in any group (*p*>0.05; Wilcoxon test).

**Figure 5:**
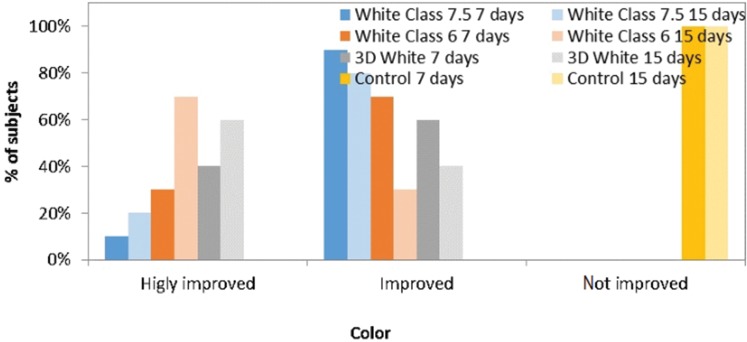
Subjects’ overall perception of improvement.

Most subjects who used whiteners found some improvement after only one week of treatment, with statistically significant differences in comparison to the control group (*p*=0.0004, *p*<0.0001 and *p*<0.0001 for White Class 7.5, White Class 6 and 3D White, respectively; Student-Newman-Keuls test). Similar results were found in the second week, in which all whitening groups differed significantly from the control (*p*=0.0017, *p*<0.0001 and *p*<0.0001 for White Class 7.5, White Class 6 and 3D White, respectively; Student-Newman-Keuls test), as shown in Figure 5. No statistically significant differences in improvement were found between the first and second week in any group (*p*>0.05; Wilcoxon test).

**Figure 6:**
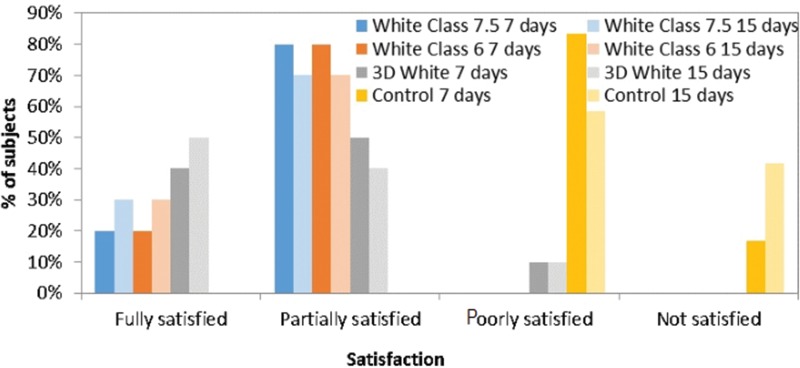
Subjects’ overall perception of satisfaction with the color.

Most subjects who used whiteners were partially satisfied with the perceived color change after only one week of whitening, with statistically significant differences in comparison to the control group (*p*=0.0001, *p*=0.0001 and *p*<0.0001 for White Class 7.5, White Class 6 and 3D White, respectively; Student-Newman-Keuls test). Similar results were found in the second week, in which all whitening groups differed significantly from the control (*p*=0.0001, *p*=0.0001 and *p*<0.0001 for White Class 7.5, White Class 6 and 3D White, respectively; Student-Newman-Keuls test), as shown in Figure 6. No statistically significant differences in satisfaction were found between the first and second week in any group (*p*>0.05; Wilcoxon test).

Regarding overall satisfaction with color rated on a scale of 0 (not satisfied) to 10 (fully satisfied), no group exhibited a significant improvement between the first and second week (*p*>0.05; Wilcoxon test). In the first week, the median overall satisfaction was similar among all treated groups and differed significantly from the control group (*p*=0.0011, *p*<0.0001 and *p*<0.0001 for White Class 7.5, White Class 6 and 3D White, respectively; Student-Newman-Keuls test). Similar results were found in the second week (*p*=0.0009, *p*<0.0001 and *p*<0.0001 for White Class 7.5, White Class 6 and 3D White, respectively; Student-Newman-Keuls test).

**Figure 7:**
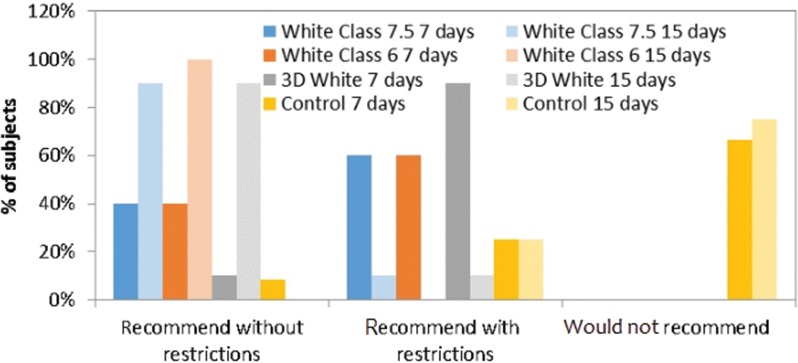
Subjects’ willingness to recommend the treatment.

All subjects who used whiteners would have recommended this process with or without restrictions after only one week of whitening, with statistically significant differences in comparison to the control group (*p*=0.002, *p*=0.002 and *p*=0.037 for White Class 7.5, White Class 6 and 3D White, respectively; Student-Newman-Keuls test). Similar results were found in the second week, in which all whitening groups differed statistically from the control (*p*=0.0001, *p*<0.0001 and *p*=0.0001 for White Class 7.5, White Class 6 and 3D White, respectively; Student-Newman-Keuls test), as shown in Figure 7. The groups did not significantly change their recommendation between the first and second week of treatment (*p*>0.05; Wilcoxon test).

## DISCUSSION

The initial and follow-up color values of the incisors and canines of the adolescents who participated in the present study were determined using the Vita Toothguide 3D-Master scale (VITA). Figure 1 shows the boxplot of the median rank colors for the White Class 7.5, White Class 6 and 3D White groups 7, 15, 30, 180 and 360 days after treatment. This figure demonstrates that all whitening agents achieved similar long-term results at the end of the study. The increase in the whitening effect after treatment may be explained by the fact that adolescents are more cooperative and motivated to control biofilm (plaque) on their incisors. Moreover, residual whitening gels can remain on the teeth for a length of time following treatment, which allows the continuation of the whitening effect even after application of the product 18. The literature shows variations in the color stability of bleached teeth, with 18 months considered the mean stabilization time 4,3,19,20. In the present study, all three products achieved excellent color stability 12 months after treatment.

The graphs in Figure 2 demonstrate that the whitening strips resulted in a faster bleaching effect in comparison to the products involving gel and a mouth tray. This may have occurred due to the greater concentration of hydrogen peroxide in the strips or the difference in consistency of the delivery medium, which can affect the diffusion of hydrogen peroxide. However, the patients who used the strips reported greater discomfort than those who used gels and mouth trays, such as increased sensitivity and a burning sensation on the gums. In a study in 2012, patients reported preferring mouth trays to strips 21.

The mouth trays were molded by dental professionals, which allowed greater control over the application of the gel with regard to respecting the limits of the gingival margin in comparison to the self-applied strips. It should be noted that the group using whitening strips was given instructions on how to apply the strips, which does not occur in real life, as this product is sold over the counter with no advice given on the best application method. A total of 30% of the patients found it hard to apply the strips, whereas patients found the trays very easy to slightly difficult to use. The ease experienced during application of the product could lead to greater harm to gingival tissues or even use by individuals for whom whitening treatment is contraindicated. Indeed, such products are often associated with gum irritation and sensitivity, even at low concentrations of peroxide 22. In the present study, the only drawback found with the tray technique was the fact that some of the patients had to remove excess gel after the procedure by rinsing.

The incisors whitened at a faster rate than the canines. This likely occurred because canines are more saturated, meaning that this type of tooth has a greater dental mass and a larger amount of extrinsic pigment 23. In a study in which the authors used 10% carbamide peroxide for 14 days, the color of the incisors stabilized in the sixth week, while the canines only stabilized in the twelfth week 24.

Since most studies have evaluated whitening procedures used by young adults rather than adolescents, the evaluation of the pulp response to this procedure is an important aspect to consider, as the component released by hydrogen peroxide can diffuse through mineralized tissues and reach the pulp chamber, thereby causing oxidative stress and permanent cytotoxic effects. Free radicals resulting from the breakdown of hydrogen peroxide, such as hydroxyl ions and reactive oxygen species, can cause the oxidation of the phospholipid chain of the cell membrane and cytoplasm lysosome, causing pulp cell damage that can range from oxidative stress to cell death 25. In a study conducted in 2010, the authors speculated that tooth whitening with gels containing a high concentration of hydrogen peroxide could cause pulp damage and pain in incisors due to the thinner enamel and dentin in comparison to posterior teeth 26. Thus, one may question the use of high-concentration whitening agents in adolescents, as this population has thinner dentinal tissue compared to individuals with more mature teeth. Therefore, at-home whitening methods with gels containing a lower concentration of hydrogen peroxide seem to be more indicated for this population. In a study published in 2015, the researchers compared the use of 6% and 35% hydrogen peroxide gels and achieved the same result at the one-month evaluation, thereby demonstrating no need to use a higher concentration 16. In contrast, the conclusion from a randomized clinical trial in adolescents conducted in 2016 was that in-office bleaching systems could be used on young permanent teeth 27.

Sensitivity and discomfort are usually more pronounced in the first week of whitening treatment 14-16, as occurred in the present study. Discomfort decreased after the second week. Gels with lower concentrations of hydrogen peroxide, such as gels used with at-home techniques, require a longer contact time with the teeth to whiten as well as gels with a higher concentration. However, gels with higher concentrations are more likely to cause sensitivity 28. In the present study, no statistically significant differences were found with regard to sensitivity in Groups 1, 2 and 3, which only differed in comparison to the control group. A previous randomized clinical trial was conducted in 2014 with four different types of at-home bleaching gels, and the authors concluded that sensitivity and intensity were not significant 29. The authors of another study 22 investigated an over-the-counter tray system and found that treatment was tolerable and safe, with a low incidence of adverse effects. Moreover, the adverse effects associated with the use of the whitening gel and tray were temporary and easily controlled. In the present investigation, sensitivity in the teeth and gums and burning on the gums/lips were reported by some of the patients, mostly by those who used strips, which is an over-the-counter product. Therefore, the use of these products without consulting a dental professional can lead to a greater risk of temporary patient discomfort.

Adolescents believe that a nice, healthy smile affects social relationships, such as dating and popularity, as well as self-esteem, obtaining good employment and future success. This population equates white teeth with healthy teeth, whereas yellowed teeth can lead to stigmas and stereotypes on the part of both those who see the teeth and those with such teeth 30,31. It is therefore important to evaluate levels of satisfaction after treatment. In the present study, most patients were partially satisfied after the first week, and this remained true for the second week. The participants also noted a difference in tooth color after the procedures. In contrast, the authors of a study conducted in 2015 evaluated the treatment time required to achieve participant satisfaction with at-home and in-office tooth whitening procedures and found that patient satisfaction took between four and six weeks regardless of the bleaching protocol employed 32. As tooth whitening is usually an elective treatment 33, it is crucial to determine patient satisfaction at the end of the treatment and whether individuals having undergone such treatment would recommend it to others. In the present study, all individuals who used the whiteners would have recommended this process with or without restrictions after only one week of whitening and continued to make the same statements after the second week. This was not true for the control group, demonstrating that the patients’ perceptions of treatment were mainly correct.

The present study is relevant when one considers the increase in the demand for tooth whitening procedures on the part of adolescents in both the dental office as well as in over-the-counter products. Few studies have addressed tooth whitening among adolescents, and further investigations are needed to determine the effects of these procedures on young teeth. Because of this, teeth whitening in adolescents should be addressed with caution. It is possible that the whitening effect is achieved at a faster rate among young patients in comparison to older patients, mainly because young teeth have fewer stains that have been on the tooth surface for a shorter period.

This study has limitations that should be addressed, such as difficulties in getting the participants to return for the follow-up assessments and the loss of adolescents due to the absence of informed consent signed by parents/guardians.

Based on the present findings, similar results were obtained after one month of treatment with tooth whitening gels at different concentrations and whitening strips. However, the whitening strips caused greater discomfort among the adolescents due to difficulties in positioning the strips correctly, leading to greater sensitivity and irritation of the gingival tissue. The patients were partially satisfied with treatment after the first and second weeks and would recommend it with or without restrictions. All products demonstrated color stability after 12 months of follow-up. Further investigations are needed to determine the effects of bleaching on young teeth.

## AUTHOR CONTRIBUTIONS

Pinto MM, Gonçalves ML, Mota AC, Olivan SR, Bortoletto C and Godoy CH helped in recruiting patients and took part in the bleaching procedures and data acquisition. Deana AM helped with the statistical analyses. Vergilio KL, Altavista OM, Motta LJ and Bussadori SK took part in writing and revising the manuscript. All authors participated in the work and take responsibility for the content.

## Figures and Tables

**Chart 1:** Summary of experimental conditions

Group	Application site of whitening gel	Self-administered treatment
1	Maxillary and mandibular incisors and canines	6.0% hydrogen peroxide White Class with calcium (FGM) in mouth trays No activating source 1 hour a day for 7 days
2	Maxillary and mandibular incisors and canines	7.5% hydrogen peroxide White Class with calcium (FGM) in mouth trays No activating source 1 hour a day for 7 days
3	Maxillary and mandibular incisors and canines	Whitening strips (Oral-B 3D White) No activating source 30 min twice a day for 7 days
4	Maxillary and mandibular incisors and canines	Placebo gel 1 hour a day for 7 days

**Table 1:** Demographic data of the groups.

Group	N	Gender		Age (years)
		Male (%)	Female (%)	Median [range]
**White Class 7.5**	**10**	**5 (50%)**	**5 (50%)**	**17.5 [14 - 25]**
**White Class 6**	**10**	**3 (30%)**	**7 (70%)**	**16.0 [13 - 19]**
**3D White**	**10**	**8 (80%)**	**2 (20%)**	**16.0 [14 - 27]**
**Control**	**12**	**8 (67%)**	**4 (33%)**	**17.0 [13 - 27]**

**Table 2:** Ranking on the Vita 3D-Master color scale.

Color	Rank
**1M1**	0
**1M2**	1
**2M1**	2
**2M2**	3
**2R1.5**	4
**2M3**	5
**3M1**	6
**3M2**	7
**3M3**	8

**Table 3:** Number of complaints after 7 and 15 days of treatment (the same subject may have had more than one complaint).

Groups	White Class 7.5		White Class 6		3D White		Control	
Parameters	7 days	15 days	7 days	15 days	7 days	15 days	7 days	15 days
Dental sensitivity	2	1	1	1	7	0	0	0
Gingival sensitivity	2	2	1	4	6	2	0	0
Itching	0	0	0	0	0	0	0	0
Mouth ulcers	0	0	0	0	3	1	0	0
Burning on lips	2	2	0	1	1	0	0	0
Burning on gums	0	0	0		1	0	0	0
Number of complaining subjects	4 (40%)	2 (20%)	1 (10%)	4 (40%)	10 (100%)	2 (20%)	0 (0%)	0 (0%)

**Table 4:** Tooth sensitivity by group.

Groups	Sensitivity							
	White Class 7.5		White Class 6		3D White		Control	
Parameter	7 days	15 days	7 days	15 days	7 days	15 days	7 days	15 days
Sensitive	50%	10%	80%	30%	80%	0%	0%	0%
Not sensitive	50%	90%	20%	70%	20%	100%	100%	100%
SD	53%	32%	42%	48%	42%	0%	0%	0%
N	10	10	10	10	10	10	12	12
